# Enhancement of Inverted Polymer Solar Cells Performances Using Cetyltrimethylammonium-Bromide Modified ZnO

**DOI:** 10.3390/ma11030378

**Published:** 2018-03-04

**Authors:** Chung-Kai Wu, Kundan Sivashanmugan, Tzung-Fang Guo, Ten-Chin Wen

**Affiliations:** 1 Department of Chemical Engineering, National Cheng Kung University, Tainan 70101, Taiwan; kai820101@gmail.com (C.-K.W.); sivashanmugannst87@gmail.com (K.S.); 2 Department of Photonics, National Cheng Kung University, Tainan 70101, Taiwan; guotf@mail.ncku.edu.tw

**Keywords:** polymer solar cells, cetyltrimethylammonium bromide, zinc oxide, power conversion efficiency, stability test

## Abstract

In this study, the performance and stability of inverted bulk heterojunction (BHJ) polymer solar cells (PSCs) is enhanced by doping zinc oxide (ZnO) with 0–6 wt % cetyltrimethylammonium bromide (CTAB) in the sol-gel ZnO precursor solution. The power conversion efficiency (PCE) of the optimized 3 wt % CTAB-doped ZnO PSCs was increased by 9.07%, compared to a PCE of 7.31% for the pristine ZnO device. The 0–6 wt % CTAB-doped ZnO surface roughness was reduced from 2.6 to 1 nm and the number of surface defects decreased. The X-ray photoelectron spectroscopy binding energies of Zn 2p^3/2^ (1021.92 eV) and 2p^1/2^ (1044.99 eV) shifted to 1022.83 and 1045.88 eV, respectively, which is related to strong chemical bonding via bromide ions (Br^−^) that occupy oxygen vacancies in the ZnO lattice, improving the PCE of PSCs. The concentration of CTAB in ZnO significantly affected the work function of PSC devices; however, excessive CTAB increased the work function of the ZnO layer, resulting from the aggregation of CTAB molecules. In addition, after a 120-hour stability test in the atmosphere with 40% relative humidity, the inverted device based on CTAB-doped ZnO retained 92% of its original PCE and that based on pristine ZnO retained 68% of its original PCE. The obtained results demonstrate that the addition of CTAB into ZnO can dramatically influence the optical, electrical, and morphological properties of ZnO, enhancing the performance and stability of BHJ PSCs.

## 1. Introduction

Bulk heterojunction (BHJ) polymer solar cells (PSCs) with an active layer composed of a conjugated polymer and a fullerene derivative as the donor and acceptor, respectively, are a promising technology for sustainable energy [1,2,3,4,5]. Such cells have received attention due to their potential for realizing lightweight, flexible, and large-area devices [6]. Impressive power conversion efficiencies (PCEs) have been reported at 10% and above [7], with extensive research conducted on device structure, material, and interface engineering. The conventional device architecture of PSCs is composed of a transparent conducting oxide (e.g., indium tin oxide, ITO) as the front anode and a low-work-function metal (e.g., Ca, Al) as the back cathode [8]. Such devices have severe stability issues resulting from the rapid oxidation of the air-sensitive cathode [9] and the corrosion of ITO due to the acidic nature of the hole-transporting layer of poly(3,4-ethylene dioxythiophene):(polystyrene sulfonic acid) (PEDOT:PSS) [10,11,12].

In order to overcome these problems, an inverted architecture was developed in 2005 [13], the active layer of which is sandwiched between an air-stable metal (e.g., Ag, Au), ITO, and further PEDOT:PSS replaced by conductive oxides, which serve as the electron and hole extraction layers. In addition, an air-stable metal can be deposited in the ambient atmosphere without a vacuum coating, simplifying and lowering the cost of manufacturing [14,15,16,17]. Although the inverted architecture can largely improve operational lifetime, PSCs based on this architecture have a trade-off between stability and performance, with PCEs usually inferior to those obtained with the conventional architecture. The performance degradation is mostly due to the poor interfacial contact between the organic active layer and the inorganic conductive oxide, which increases the number of surface trap states and hinders the electron extraction ability of electron extraction layers (EELs), which further decreases short-circuit current density (*J_sc_*) and the fill factor (FF) [18,19,20,21].

Titanium oxide (TiO_x_) [22,23,24,25], zinc oxide (ZnO) [26,27], and aluminium oxide (Al_2_O_3_) [28] are the most widely used materials for the conductive oxide serving as the EEL in the inverted architecture due to their high device performance and exceptional air stability. Among them, ZnO possesses several remarkable features, such as high transparency, high conductivity, low cost, and facile solution processing, including that conducted using atomic layer deposition [29], nanoparticle (NP) approaches [30,31,32,33,34,35,36], and sol-gel deposition [20,27,37,38]. Furthermore, Zn is non-toxic and abundant. Despite the facile processing of ZnO NPs via spin-coating or roll-to-roll printing at room temperature, the obtained NPs are not stable. A ligand is thus usually used to stabilize them [39]. In contrast, sol-gel-processed ZnO can be obtained at lower annealing temperature (200 °C) [27] and serves as a stable EEL. Nevertheless, considerable improvement in device performance is needed due to the imbalance of electron and hole carriers. Moreover, low-temperature-annealed ZnO commonly has numerous surface defects. These defects act as recombination centers for charge carriers, lowering PCE.

Modification of the interface between the organic semiconductor and the inorganic electrode can be used to adjust the bandgap of each contact to achieve ohmic contact. The work function between the contact layers greatly affects the carrier injection and transport in organic semiconductors. Interfacial modification can also greatly improve the surface morphology of a metal oxide EEL, further enhancing the *J_sc_* and FF of a device. Approaches such as inserting a thin polymer layer [40,41,42], fullerene derivatives [43], polyelectrolytes [44], and zwitterions [45], as well as doping the EEL [46,47] have been investigated. Polymer materials can be classified into polyelectrolytes and insulating polymers. A previous study found that the polyelectrolyte poly[3-(6-trimethylammoniumhexyl)thiophene] (P3TMAHT), which is composed of a conjugated backbone and a functional group of quaternary ammonium salts, can induce interfacial dipoles between the cathode and the active layer, efficiently increasing the *J_sc_* and open-circuit voltage (*V_oc_*) of a device [48]. Insulating polymers such as polyethyleninmine (PEI), polyethylenimine ethoxylated (PEIE), polyethylene oxide (PEO), and polyvinylpyrrolidone (PVP) have a lower price. The amine functional groups on PEI and PEIE can spontaneously form intramolecular and interfacial dipoles, decreasing the work function of ZnO [40,49,50]. However, amine functional groups-based materials are very expensive and unstable, which are greatly affected by the synthesis technology. The use of small-molecule materials with a simple structure and facile purification leads to better reproducibility. 

**Figure 1:**
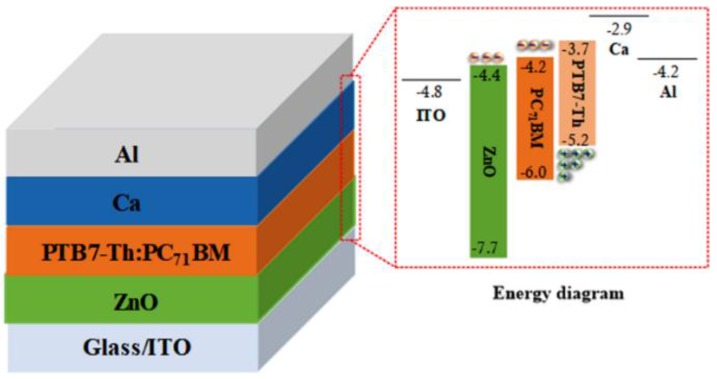
Conventional architecture of ZnO-based BHJ PSCs and corresponding energy diagram.

Wen et al. applied a small-molecule material, namely tetraoctylammonium bromide (TOAB), as the EEL in a polymer solar cell. TOAB can establish a self-assembled monolayer (SAM) on the polymer active layer, enhancing the PCE of the device from 2.38% to 4.19% [51]. These results demonstrate that modification with quaternary ammonium salts is efficient even without the conjugated backbone of conjugated polyelectrolytes. Yu et al. utilized 1-butyl-3-methylimidazolium tetrafluoroborate ([BMIM] BF4), an ionic liquid, in an inverted system with PTB7-Th:PC_71_BM, which enhanced the PCE to 10.15% by reducing the interfacial barrier. [BMIM]BF4 spontaneously induced interfacial dipoles with the different levels of hydrophilic between the ZnO and active layers. They remedied the poor interfacial contact between the inorganic ZnO EEL and the organic polymer active layer while maintaining the high stability of an inverted structure. In the present work, we investigate a ZnO interfacial layer modified with quaternary ammonium salts, namely cetyltrimethylammonium bromide (CTAB), in PSCs (Figure 1). The ionic pendant groups ensure solubility in polar solvents, allowing the solution-based fabrication of multilayer devices and improving the PCE of PSCs.

## 2. Materials and Methods

### 2.1. ZnO Precursor Solution Preparation

The ZnO precursor solution was prepared by dissolving zinc acetate dihydrate (99.5%, 1 g Merck, Kenilworth Fort, NJ, USA) and ethanolamine (98%, 0.28 g, Acros, Geel, Belgium) in 2-methoxyethanol (98%, 10 mL, Sigma-Aldrich, Saint Louis, MO, USA) under stirring for 8 h for a hydrolysis reaction. Then, 0–6 wt % CTAB and R6G were added to the ZnO precursor solution, respectively. The as-prepared ZnO precursor solution was further used for device fabrication. 

### 2.2. Fabrication of BHJ PSCs

Indium tin oxide (ITO) glass substrates (sheet resistance: < 15Ω/sq) were purchased from RITEK Corp (Hsinchu, Taiwan). The ITO substrates were cleaned ultrasonically with detergent, deionized water, acetone, and isopropyl alcohol in sequence for 15 min each. Subsequently, they were treated with oxygen plasma for 25 min. A ZnO precursor solution was spin-coated onto pre-cleaned ITO-coated glass substrates at 4000 rpm for 60 s and then annealed at 200 °C for 1 h in air to form a thin layer of ZnO (≈30 nm). The active layers (PTB7-Th:PC_71_BM) (≈90 nm) were obtained by spin-coating the active layer solution, which was filtered using a polytetrafluoroethylene filter (0.45 μm), atop the thin layer of ZnO. The anode of each device, MoO_3_/Ag (3 nm/80 nm), was thermally evaporated at a pressure of ≈10^−7^ Torr. The area of each device was 6 mm^2^, as defined by a shadow mask. The *J*-*V* characteristics of devices under AM 1.5G illumination at 100 mW·cm^−2^ were measured using a Keithley 2400 source-measure (Cleveland, OH, USA) unit in a nitrogen-filled glove box at room temperature. All devices were measured without any shadow masking. The AM 1.5G illumination was simulated using an Oriel 300-W Solar Simulator (Newport Company, Irvine, CA, USA) and calibrated using a silicon photodiode with a protective KG5 filter calibrated by the National Renewable Energy Laboratory (Denver, CO, USA). The morphology, chemical composition, and optical properties of as-fabricated devices were examined using scanning electron microscopy (SEM, JSM-7001, JEOL, Tokyo, Japan) with energy-dispersive X-ray spectroscopy (EDS) (JSM-7001, JEOL, Tokyo, Japan), atomic force microscope (AFM, Dimension Icon, Bruker, Billerica, MA, USA), photoluminescence (PL) (Renishaw, UK) and ultraviolet-visible (UV-Vis) spectrophotometry (HITACHI U4100, Tokyo, Japan). The surface composition of the pristine and surface modified devices were analyzed using X-ray photoelectron spectroscopy (XPS) with an Al Kα (1486.6 eV) X-ray source (Microlab 350, Thermo Fisher Scientific, UK). 

## 3. Results and Discussion

**Figure 2:**
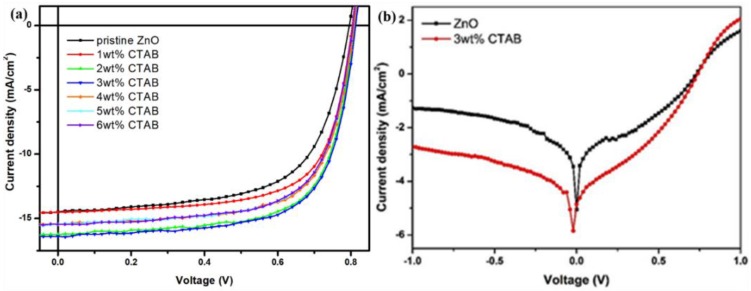
(**a**) *J*-*V* curve of devices based on CTAB-doped ZnO and (**b**) dark *J*-*V* curves of devices based on pristine ZnO and 3 wt %-CTAB-doped ZnO. Devices were examined under AM 1.5G illumination at 100 mW·cm^−2^.

The configuration of devices was ITO/CTAB-doped ZnO/PTB7-Th:PC_71_BM/Ca, MoO_3_/Ag, or Al, as shown in Figure 1. Devices with a configuration of ITO/ZnO/PTB7-Th:PC_71_BM/Ca/Al were also fabricated for reference. The photovoltaic performance of as-fabricated CTAB-doped ZnO devices was compared with that of a pristine ZnO device. Figure 2a,b shows the current density-voltage (*J*-*V*) characteristics of devices with various concentrations (0–6 wt %) of CTAB doped into ZnO evaluated under air mass 1.5 global (AM 1.5G) illumination at 100 mW·cm^−2^ and in the dark, respectively. The detailed device performance parameters are listed in Table 1. The PCE of as-fabricated devices increased from 7.31% to 9.07% after doping with CTAB, which can be ascribed to better electronic coupling at the interface between the organic conductive oxide and the organic active layer. The *V_oc_* of devices slightly increased, which is attributable to the increased built-in potential across devices after doping with CTAB and decreased work function of ZnO. The decrease in work function is supported by the dark *J*-*V* characteristics shown in Figure 2b; the diodes turn on at a voltage at which the built-in potential is compensated. The series resistance (R_S_) was affected by the decrease in work function; it was 145.59 Ω for the pristine ZnO device and 102.94 Ω for the 2 wt %-CTAB-doped ZnO device. This may indicate that CTAB-doped ZnO decreases the contact resistance at the ZnO and PTB7-Th:PC_71_BM interface, which is favorable for electron extraction. Therefore, devices with an optimized concentration (3 wt %) of CTAB doped into ZnO showed a larger *J_sc_* than that of the device with pristine ZnO. 

**Figure 3:**
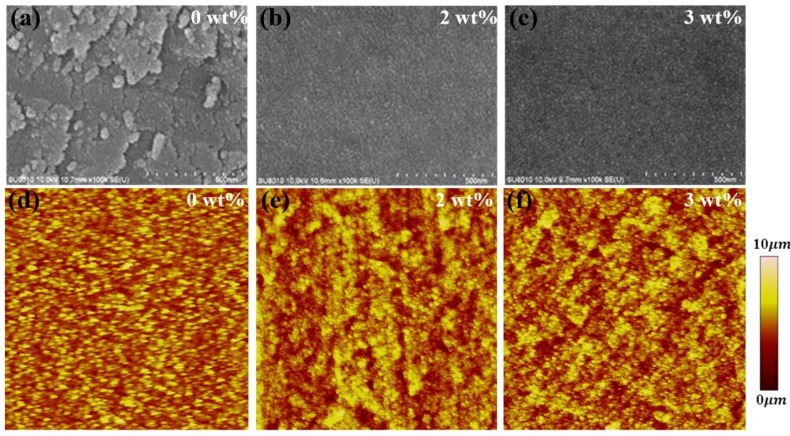
SEM and AFM images of devices based on (**a**, **d**) 0 wt %-, (**b**, **e**) 2 wt %-, and (**c**, **f**) 3 wt %-CTAB-doped ZnO.

Figure 3 shows top-view SEM and AFM images of pristine ZnO and CTAB-doped ZnO devices. The CTAB-doped ZnO surface has an improved morphology compared with that of pristine ZnO. The pristine ZnO surface is the roughest, and is composed of many discrete and irregular grains; after the ZnO surface incorporates CTAB, it becomes smooth and the number of defects is reduced. Further increasing the concentration of CTAB on the ZnO surface led to the formation of large clusters due to CTAB aggregation. Figure 3d–f shows the AFM tapping mode topography of pristine ZnO and CTAB-doped ZnO devices. The roughness (R_a_) of the ZnO surface decreased from 2.6 (pristine) to 1.05 nm (3 wt %) after doping with CTAB. However, excessive CTAB slightly increased R_a_, but the surface was still smoother than that of pristine ZnO. The results show that the morphology of CTAB-doped ZnO was greatly improved, which decreased the leakage current and increased FF. Moreover, with the combined effect of increased *V_oc_*, *J_sc_*, and FF, devices with CTAB-doped ZnO had improved photocurrent and charge selectivity during the charge transfer process and exhibited higher PCE values compared with those of the pristine-ZnO-based device.

**Figure 4:**
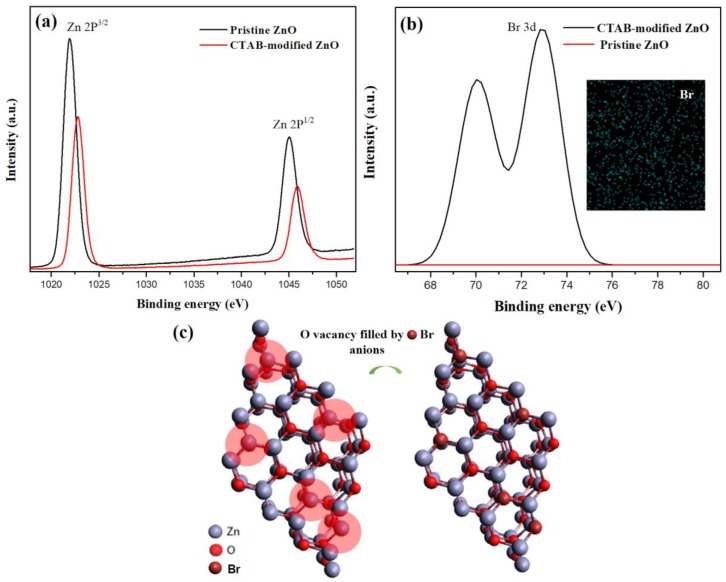
(**a**) Zn 2p and (**b**) Br 3d XPS spectra of device based on CTAB-doped ZnO. Inset EDS image in (**b**) shows Br distribution on device with CTAB-doped ZnO. (**c**) Illustration of crystal structure, showing Br anions occupying O vacancies in ZnO after CTAB doping.

Figure 4a shows XPS spectra of pristine ZnO and CTAB-doped ZnO layers. The characteristic peaks of Zn 2p^3/2^ and 2p^1/2^ were at 1021.92 and 1044.99 eV, respectively, indicating pristine Zn. After CTAB doping, the Zn peaks were at (Zn 2p^3/2^) 1022.83 and (Zn 2p^1/2^) 1045.88 eV, indicating shifts compared to those for pristine Zn. In addition, the XPS peak intensity of CTAB-modified ZnO decreased due to the strong binding of CTAB on the ZnO surface. The XPS peaks of Br 3d for the CTAB-doped ZnO surface were at 70.06 and 72.92 eV, which confirms Br^−^ on the ZnO surface, as shown in Figure 4b. Moreover, the EDS mapping shows Br uniformly distributed in the ZnO crystal, as shown in the inset in Figure 4b, where a content of 64.45 wt % Zn, 25.77 wt % O, and 9.78 wt% Br was measured. These results confirm that CTAB was present in the ZnO crystal structure and that it affected the properties of ZnO. The shift of the Zn 2p orbital peak can be attributed to the formation of chemical bonding between Zn and Br. We suspect that the bromide anions of CTAB occupy the oxygen vacancies in the ZnO crystal Figure 4c, which is indicated small area of ZnO crystal structure affected by the defects due to natural formed oxygen vacancies in lattice. When anions bond with Zn, electrons are released, further increasing the conductivity of ZnO. 

**Figure 5:**
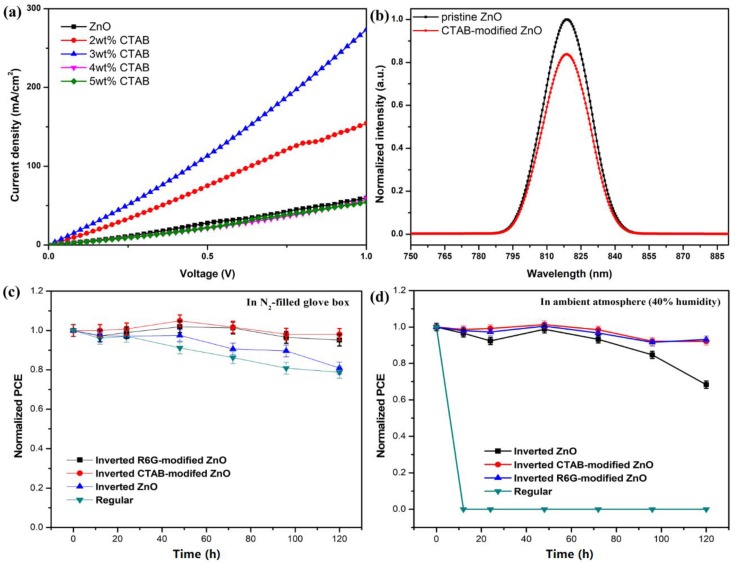
(**a**) *J*-*V* curve of electron-only device with structure of ITO/ZnO/PTB7-Th:PC_71_BM/Ca/Al. (**b**) PL spectra of devices based on pristine ZnO and CTAB-doped ZnO. Stability studies of devices based on CTAB-doped ZnO in (**c**) N_2_ atmosphere and (**d**) ambient atmosphere.

To confirm the enhancement in the electron extraction ability of the CTAB-doped ZnO device, we fabricated an electron-only device, in which the hole-transporting MoO_3_ and Ag electrode were replaced by Ca and an Al electrode (i.e., the device architecture is ITO/ZnO/PTB7-Th:PC_71_BM/Ca/Al). From the *J*-*V* curve of the electron-only device (Figure 5a), 3 wt %-CTAB-doped ZnO shows the best electron extraction ability, which is substantially improved over that of the pristine ZnO. The device current density is mainly attributed to electrons because the energy barrier is enormous for the holes, which means that the holes cannot transport to external circuit. Figure 5b shows the PL spectra of CTAB-doped ZnO. The PL spectra of CTAB-doped ZnO has lower intensity than that of pristine ZnO, indicating a reduction in the charge recombination loss and showing that CTAB-doped ZnO has better electronic coupling capability. 

Finally, stability tests were performed in both an ambient atmosphere and a nitrogen-filled glovebox for up to 120 h; the results are shown in Figure 5c. After 120 h in the nitrogen-filled glove box, the CTAB-doped ZnO device retained 98% of its original PCE; the pristine ZnO device retained 81% of its original PCE. The conventional device (ITO/PEDOT:PSS/PTB7-Th:PC_71_BM/Ca/Al) retained just 78% of its original PCE. In the ambient atmosphere, the conventional device is totally devastated in the first 12 h; the CTAB-doped ZnO device retained 92% of its original PCE, and the pristine ZnO device retained 68%, as shown in Figure 5d. In addition, rhodamine 6G (R6G) modified ZnO surface in PSCs was used as reference device, whereas device performance and stability improved as compared to pristine ZnO. The ZnO surface became more hydrophobic after CTAB doping, which is related to the surface distribution of the long alkyl chain of CTAB. The formation of an hydroxide phase on the ZnO surface was significantly controlled by CTAB.

## 4. Conclusions

This study demonstrated a facile approach for passivating defects in the ZnO EEL and improving the interfacial contact in an inverted architecture simply by doping CTAB into the sol-gel ZnO precursor. This improves the morphology of ZnO and leads to better contact by increasing hydrophobicity. These improvements resulted in better electron extraction ability, enhancing the performance of BHJ PSCs. The PCE of the optimized 3 wt % CTAB-doped ZnO PSCs was achieved 9.07%. With incorporation of CTAB into ZnO, the PCE increased by 24% compared to that obtained for pristine ZnO as the EEL.

## Figures and Tables

**Table 1:** Detailed performance parameters of devices based on CTAB-doped ZnO.

EEL	*V*_OC_ (V)	*J*_SC_ (mA/cm^2^)	FF (%)	PCE (AVG) (%) *	*R*_S_ (Ω)
Pristine ZnO	0.80	14.49	63.03	7.31	145.59
1 wt % CTAB	0.81	14.52	67.66	7.93	139.34
2 wt % CTAB	0.82	16.23	67.65	8.99	102.94
3 wt % CTAB	0.82	16.38	67.51	9.07	105.90
4 wt % CTAB	0.81	15.47	67.62	8.48	148.26
5 wt % CTAB	0.81	15.45	66.97	8.36	138.75
6 wt % CTAB	0.81	15.44	66.82	8.34	140.53

* The presented averaged PCE values were averaged from 25 devices.
